# Improved Serodiagnosis of Cystic Echinococcosis Using the New Recombinant 2B2t Antigen

**DOI:** 10.1371/journal.pntd.0001714

**Published:** 2012-07-03

**Authors:** Ana Hernández-González, Saúl Santivañez, Héctor H. García, Silvia Rodríguez, Santiago Muñoz, Guillermo Ramos, Antonio Orduña, Mar Siles-Lucas

**Affiliations:** 1 IRNASA, CSIC, Salamanca, Spain; 2 Instituto Peruano de Parasitología Clínica y Experimental (INPPACE), Lima, Perú; 3 Hospital Clínico de Salamanca, Salamanca, Spain; 4 Hospital Universitario de Valladolid, Valladolid, Spain; Queensland Institute of Medical Research, Australia

## Abstract

A standardized test for the serodiagnosis of human cystic echinococcosis (CE) is still needed, because of the low specificity and sensitivity of the currently available commercial tools and the lack of proper evaluation of the existing recombinant antigens. In a previous work, we defined the new ELISA-B2t diagnostic tool for the detection of specific IgGs in CE patients, which showed high sensitivity and specificity, and was useful in monitoring the clinical evolution of surgically treated CE patients. Nevertheless, this recombinant antigen gave rise to false-negative results in a percentage of CE patients. Therefore, in an attempt to improve its sensitivity, we constructed B2t-derived recombinant antigens with two, four and eight tandem repeat of B2t units, and tested them by ELISA on serum samples of CE patients and patients with related parasites. The best diagnostic values were obtained with the two tandem repeat 2B2t antigen. The influence of several clinical variables on the performance of the tests was also evaluated. Finally, the diagnostic performance of the 2B2t-ELISA was compared with that of an indirect haemagglutination commercial test. The 2B2t recombinant antigen performed better than the HF and B2t antigens, and the IHA commercial kit. Therefore, this new 2B2t-ELISA is a promising candidate test for the serodiagnosis of CE in clinical settings.

## Author Summary

Cystic echinococcosis (CE) is a widespread zoonotic disease. Its complex clinical presentation precludes a “one-size-fits-all” approach to clinical management, particularly with regard to serodiagnosis. While CE is often detected incidentally by imaging, imaging findings may be inconclusive. Therefore, there is a need for standardised and approachable diagnostic tools that may complement imaging data. In this regard, serological tests based in the use of native antigens like hydatid fluid present a low specificity and sensitivity. Although recombinant antigens with potential to replace native antigens have been proposed in the literature, none has been systematically tested for diagnostic performance. Here, we describe the new recombinant antigen 2B2t, derived from the previously described recombinant B2t, and determine its usefulness for the serodiagnosis of CE by ELISA in patients with a complete set of clinical data. The influence of clinical variables on the performance of 2B2t was evaluated and compared with the hydatid fluid (the most commonly used antigen for CE serology) and a commercial diagnostic kit based on the haemagglutination reaction. Our results show that the 2B2t antigen has potential to be routinely used for the standardised diagnosis of CE in clinical settings.

## Introduction

Cystic echinococcosis (CE) is a zoonosis caused by the metacestode of *Echinococcus granulosus*. In Europe, this cestode is more commonly found in Mediterranean countries, where it is usually maintained through a domestic life cycle involving dogs and sheep [1]–[2]. CE is still a cause for concern in many European countries [3]. It is also a major problem in developing countries, where it carries significant morbidity and mortality in both humans and animals [4]–[5].

CE diagnosis is mostly based on imaging techniques and can be facilitated by using the WHO international classification of ultrasound images in CE [6]. This classification is also useful in determining the type of cyst according to its activity (i.e., active: CE1, CE2; transitional: CE3a, CE3b; or inactive: CE4, CE5), and to monitor the clinical evolution of patients. Most importantly, the classification was recently validated by a study that used Magnetic Resonance Spectroscopy to investigate the metabolic profiles of the different cyst stages [7]. Laboratory and imaging data are usually sufficient to establish a reliable diagnosis but sometimes they are inconclusive [8]. An immunoassay can be used as a confirmatory test, and usually consists of an *E. granulosus* hydatid fluid (HF) antigen ELISA [8]. The sensitivity of the HF-ELISA for the diagnosis of different cases of CE ranges between 50 and 98%, and depends on the localization, size, number and stage of cysts. Several other factors, such as the time between initiation of treatment onset and the date of serum collection, CE antecedents (patients suffering of a previous CE), and the presence of complications, could also affect the results of the tests. This may explain the great variability in the sensitivity reported by different laboratories using the same antigen (HF or recombinant antigens) [9]. However, in most articles published in the field, data on these variables were not reported, which prevented the development of a routine serodiagnostic tool with a consistent and clinically acceptable diagnostic performance.

Several recombinant antigens have shown potential for CE serodiagnosis [9]. In this regard, we recently proposed the use of a C-terminal truncated recombinant antigen B2 (B2t) for the diagnosis and monitoring of CE patients by ELISA. Indeed, this B2t-ELISA showed excellent diagnostic accuracy (91.2% sensitivity and 93% specificity), and had the potential to signal cure in surgically treated CE patients [10]. This study was performed on CE patients with no indication of the above-mentioned variables, except for cyst localization.

Several studies, such as the recent one by Valiente-Gabioud *et al.* [11] on a specific *Trypanosoma cruzi* antigen, showed that an increase in the number of repetitive units of an antigen could result in an enhanced antigenic response. Because the use of the B2t antigen gave rise to some false-negative results, we thought to use the same antigen, but with a variable number of tandem repeats. Therefore, we collected sera from several CE patients, as well as complete information on the variables listed above with the potential to affect the results of the tests. The capacity of the recombinant antigens obtained to diagnose CE was assessed by ELISA and compared with each other and with the HF antigen. Finally, the diagnostic performance of the new antigens was compared to that of their corresponding commercial indirect haemagglutination (IHA) kit, using serum from the same CE patients.

## Methods

### Antigens

Crude sheep HF collected from fertile hydatid cysts and containing viable protoscoleces was kindly provided by S. Jiménez (Servicio de Seguridad Alimentaria y Sanidad Ambiental, Consejería de Salud de La Rioja, Spain). The HF was centrifuged at 1,000 g for 5 min, and the protein concentration in the supernatant was measured with the Micro BCA Protein Assay Kit (Pierce). The supernatant was then stored at −80°C until use.

The B2t recombinant antigen was obtained as described before [10]. Briefly, the coding sequence of antigen B2 (GenBank entry number U15001) was cloned in the pGEX-4T2 expression vector, excluding the region coding for the signal peptide (GE Healthcare Life Sciences). This construct was then used to transform BL21-CodonPlus-RIL competent cells, derivative of *Escherichia coli* (Stratagene). Protein expression and purification were carried out as described before [10].

Three molecules with a variable number of tandem repeats (2B2t, 4B2t and 8B2t containing two, four and eight B2t subunits, respectively) were synthesised based on the original B2t recombinant antigen, as described before [12], albeit with minor modifications. Briefly, a pair of PCR primers spanning the whole B2t-pGEX-4T2 sequence (except for the terminal codon) were designed. They also included restriction sites for *Bgl*II and *Bam*HI in the 5′- and 3′-ends, respectively, which are marked in italics in the following sequences: forward primer 5′- AC*AGATCT*AAAGATGAGCCAAAAGC and reverse primer 5′- AT*GGATCC*CTTTGAATCATCATC. The PCR was carried out in a Techne T-512 termocycler, for 35 cycles. The five first cycles consisted of 40 sec at 94°C, 40 sec at 48°C and 1 min at 72°C, and the next 30 cycles consisted of 40 sec at 94°C, 40 sec at 58°C and 1 min at 72°C. The PCR product was then purified with the Strataprep DNA Gel Extraction Kit (Stratagene) and inserted into the pGEM-T Easy vector (Promega), which contains a restriction site for *Sac*I, downstream of the cloning site. Subsequently, the recombinant vector was subjected to two double digestions in parallel, one with *Bam*HI and *Sac*I, resulting in the linearisation of the construct, and another one with *Bgl*II and *Sac*I, to obtain the fragment corresponding to the B2t coding sequence. Both products were then ligated, which led to the ligation of the *Bgl*II and *Bam*HI restriction sites, and resulted in the loss of the restriction sites ligating the 2B2t molecules. This step was repeated to obtain the 4B2t and 8B2t molecules. The resulting tandem repeat constructs of 446, 874 and 1,738 base pairs, respectively, were sequenced to check for ligations and open reading frames. The three constructs were then excised from the pGEM-T Easy vector with *Eco*RI and subcloned in the pGEX-4T1 expression vector. Expression and purification of the corresponding proteins was performed as described before [10]. The purity and yield of each protein obtained after purification and thrombin cleavage of the respective GST-fused proteins were assessed in 12% polyacrylamide gels using Coomasie blue staining. The densitometry was calculated with the ImageJ software (http://rsbweb.nih.gov/ij/).

### Ethics statement

For this study, the serum of CE patients and healthy donors was used (see next section). The study was approved by the ethics committees of the University Hospitals of Salamanca and Valladolid, Spain, and the main IRB of Cayetano Heredia University of Lima, Peru.

All subjects gave their informed written consent, which specified that these samples could be used for future analyses.

### Serum

103 Spanish sera were used to test the diagnostic value of the three tandem repeat B2t recombinant proteins, and to compare it to that obtained with the original B2t and HF antigens. The individuals comprised 49 healthy donors and 54 patients with surgically confirmed CE, selected for their previously known reactivity to the B2t recombinant antigen by ELISA (12 B2t-negative and 42 B2t-positive; unpublished data).

Further characterization of the recombinant B2t and 2B2t antigens was performed using the serum of 186 Peruvian patients with CE confirmed surgically and/or by imaging. The clinical characteristics of the patients are presented in Table 1. They were recruited in the INPACCE (Lima, Peru) from 2007 to 2008, if they had clinical findings compatible with CE and were living in endemic (34%) and non-endemic (66%) areas for CE. Additionally, the serum of 110 healthy donors, 36 patients with hepatitis (both groups recruited in the Hospital of Salamanca, Spain) and 138 patients with related parasites confirmed by microscopy or specific ELISA or immunoblot assay (70 patients with neurocysticercosis, including 57 Peruvian patients with neurological symptoms and living in endemic areas for cysticercosis recruited in the Institute for Neurological Science (Lima, Peru) in 2007 and 13 Mexican patients; 57 Swiss patients with alveolar echinococcosis; and 11 Peruvian patients with taeniosis) were tested.

**Table 1:** Characteristics of the CE patients from Peru.

**CE diagnosis**	
Surgery	134 (72.0%)
Imaging	52 (28.0%)
**Gender**	
Male	85 (45.7%)
Female	101 (54.3%)
**Number of cysts**	
Single	108 (58.1%)
Multiple	78 (41.9%)
**Cyst localisation**	
Liver	65 (34.9%)
Lung	86 (46.2%)
Liver plus lung	24 (12.9%)
Other	11 (6.0%)
**Cyst classification** *	
CE1	44 (49.4%)
CE2	22 (24.7%)
CE3	8 (9.0%)
CE4	2 (2.2%)
NS	13 (14.7%)
**Complicated CE** #	
No	87 (46.8%)
Yes	98 (52.7%)
NS	1 (0.5%)
**First CE**	
Yes	20 (10.7%)
No	166 (89.3%)
**Serum collection**	
Before treatment	129 (69.3%)
After treatment	57 (30.7%)

Data are shown as total number and percentage (%) for each subgroup.

*: For liver cysts only, according to the WHO classification (2003).

# According to Beggs (1985). NS: not stated.

### Enzyme-linked immunosorbent assay (ELISA) and indirect haemagglutination test (IHA)

The sera and antigens were tested by IgG ELISA, as described before [10]. Briefly, 96-well polystyrene plates (Corning, Madrid, Spain) were coated overnight at 4°C with the B2t, 2B2t, 4B2t or 8B2t recombinant proteins (0.5 µg/ml) or with HF (5 µg/ml) in carbonate buffer, pH 9.6 (100 µl/well). The plates were then washed six times with phosphate buffered saline (PBS, pH 7.4) plus 0.05% Tween 20 (washing buffer) and incubated for 1.5 h at 37°C with 200 µl of washing buffer plus 1% bovine serum albumin (BSA; Sigma Aldrich, Madrid, Spain) (blocking buffer). Then, the sera were added in duplicate (100 µl/well) at 1∶200 dilution in blocking buffer, and the plates were incubated for 1 h at 37°C. The plates were washed again, and the secondary antibody (peroxidase-labelled rabbit anti-human IgG; Sigma Aldrich, Madrid, Spain) was added at a 1∶2000 dilution in blocking buffer (100 µl/well), and incubated for 1 h at 37°C. After washing, the plates were stained with 100 µl/well of citrate buffer pH 5 plus orthophenylene diamine (0.28 mg/ml; Sigma Aldrich, Madrid, Spain) and hydrogen peroxide (0.4 µl/ml; Sigma Aldrich, Madrid, Spain). The reaction was stopped with 50 µl/well of 3 N sulfuric acid, and the plates were read at 492 nm with an ELISA reader instrument (STL 340 ATC reader, Lab Instruments, Salzburg, Germany).

The IHA kit (Hydatidose Fumouze Kit, Fumouze Diagnostics, Levallois-Perret, France) was applied to the above-mentioned sera, according to the manufacturer's instructions. Briefly, the sera were diluted at 1∶40 in the serum diluter from the kit. This solution was used to obtain two-fold serial dilutions of each serum, as indicated by the supplier, in round-bottom plates. Then, a drop of sensitized red cells was added to each well, and the plates were incubated at room temperature for two hours. Subsequently, the agglutination reactions were interpreted according to the manufacturer's instructions. Thus, the sera were considered positive when agglutination was observed at ≥1∶320 dilutions (cut-off value of 1∶320). An agglutinin control was performed for several randomly selected sera, as recommended by the manufacturer.

### Statistics

The serological activity index was calculated for each optical density and used to establish a common cut-off value for all the ELISAs performed for each antigen, using the following formula: [(*NC*−*S*)/(*NC*−*PC*)]*100, where *NC* and *PC* are the negative and positive controls, respectively, and *S* stands for each serum. The best cut-off value was then determined by receiver operator characteristic (ROC) analysis for each antigen. For the determination of that value, sera from the 186 Peruvian CE patients were considered true positive and 110 sera from donors plus 174 sera from patients with other diseases were considered true negative.

A chi-square test was used to compare the sensitivity of the B2t, 2B2t and HF antigens for the detection of total IgG by ELISA, in groups of CE patients with different clinical and cyst-related characteristics, which could influence the resulting sensitivity. A *P* value<0.05 was considered statistically significant. Additionally, two different statistical approaches were used to evaluate the influence of the different clinical variables on the diagnostic performance of the different tests. First, a bivariate binary logistic regression was performed to calculate the odd ratio (OR) for each pair of clinical variables, and identify those associated with test performance. Then, the significant variables were further analysed by multivariate binary logistic regression, to identify those independently associated with the performance of each serological test, taking into account the simultaneous participation of the other variables. All statistical analyses were performed with SPSS v.19 (www.ibm.com).

## Results

### Recombinant antigens

**Figure 1:**
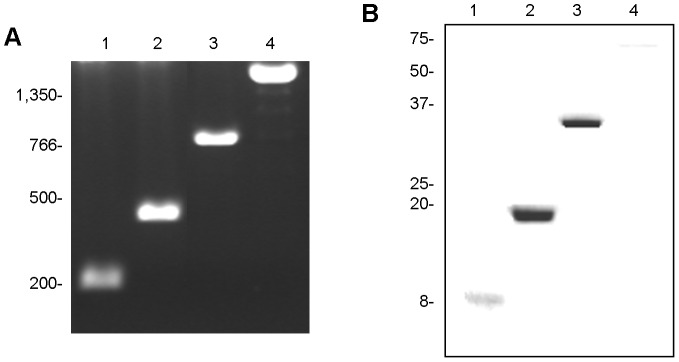
Cloning and production of the B2t (1), 2B2t (2), 4B2t (3) and 8B2t (4) recombinant proteins. (A) 1% agarose gel stained with ethidium bromide and (B) 15% acrylamide gel stained with Comassie blue, showing the polynucleotides and purified recombinant proteins. The molecular weights are indicated in base pairs (A) and kilodaltons (B): (1) B2t 213 pb, 8.3 kDa; (2) 2B2t, 446 pb, 16.9 kDa; (3) 4B2t, 874 pb, 31.3 kDa and (4) 8B2t, 1,738 pb, 60.1 kDa. In (B), 10 µl of the supernatant of each recombinant protein purified from 1 litre of culture was loaded to compare the final concentration of each recombinant protein obtained from the same volume of culture.

The tandem ligation of the B2t nucleic acid sequence of 213 base pairs (bp) resulted in three products of 446, 874 and 1,738 bp, corresponding to the 2B2t, 4B2t and 8B2t antigens, respectively (Figure 1A). The B2t, 2B2t, 4B2t and 8B2t recombinant proteins were obtained as described previously [10], by thrombin cleavage of the respective GST-fused products, resulting in polypeptides with molecular weights of 8.3, 16.9, 31.3 and 60.1 kDa, as expected, and with high purity and no degradation (Figure 1B). After purification (Fig. 1B), the highest yield was obtained for the 2B2t, followed by 4B2t, B2t and 8B2t proteins. The production of the 8B2t protein was too low and was therefore disregarded.

The 2B2t recombinant antigen was patented (P201030983).

### Usefulness of the B2t-derived recombinant antigens for the serodiagnosis of CE

#### Comparison with a commercial IHA kit

The preliminary assessment of the diagnostic performance of the recombinant B2t, 2B2t and 4B2t proteins ELISA was performed using the serum of 49 healthy donors and 54 patients with confirmed CE, selected for their previously known reactivity to the B2t recombinant antigen. As shown in Table 2, the highest sensitivity was obtained with the 2B2t antigen (92.6%), followed by the HF (81.5%) and the 4B2t antigens (79.6%). Both the 2B2t and 4B2t antigens showed a higher sensitivity than the B2t antigen in this analysis (77.8% sensitivity). The specificity obtained in the serum of the 49 healthy donors with the 4B2t antigen was only of 79.6%, and was thus lower than the specificity obtained with the HF (93.9%) and the two other recombinant antigens (95.9%). The difference in sensitivity observed between the 2B2t antigen and the HF, B2t and 4B2t antigens was statistically significant in each case. However, the difference in specificity was only statistically significant when comparing the 2B2t antigen with the 4B2t antigen (see Table 2).

**Table 2:** Comparative analysis of the diagnostic performance of four different antigens in ELISA.

	CE Confirmed cases, n = 54 (Sensitivity)			Healthy donors, n = 49 (Specificity)
	Overall (n = 54)	B2t negative (n = 12)	B2t positive (n = 42)	
**HF**	44 (81.5%*)	4 (33%)	40 (95.2%)	46 (93.9%)
**B2t**	42 (77.8%*)	0 (0%)	42 (100%)	47 (95.9%)
**2B2t**	50 (92.6%)	9 (75%)	41 (97.6%)	47 (95.9%)
**4B2t**	43 (79.6%*)	3 (25%)	40 (95.2%)	39 (79.6%*)

The antigens tested were: hydatid fluid (HF), B2t, 2B2t and 4B2t recombinant proteins. IgG specific antibodies were investigated in patients with cystic echinococccosis (CE) and healthy donors. n = number of samples.

*: indicates significant differences between the HF, B2t or 4B2t and 2B2t-ELISA.

Because of its low specificity, the 4B2t antigen was discarded from further studies, and the subsequent analyses thus focused on the B2t and 2B2t antigens.

Then, the serum of 186 Peruvian patients with confirmed CE, 110 healthy donors and 174 patients with different infectious diseases was used to compare the usefulness of the 2B2t recombinant antigen with that of the B2t and HF antigens by ELISA and by IHA with a commercial kit (Fumouze Diagnostics, Levallois-Perret, France), based on the detection of specific serum antibodies against a purified fraction of the *E. granulosus* antigen 5.

The results obtained with the 186 Peruvian CE patients are shown in Table 3, specifically sensitivities of 83.3%, 79% and 87.6% for the HF, B2t and 2B2t antigens, respectively, and of 34.9% were found when using the IHA kit. For the 2B2t-ELISA, only 5.9% of the sera from CE patients was close (±5 SI units) to its cut-off value. The difference in sensitivity between the 2B2t-ELISA and each of the other three tests was statistically significant. The evaluation of the four diagnostic tools using serum from healthy donors showed that the IHA was the most specific test, followed by the B2t- and 2B2t-ELISA, and the HF-ELISA (Table 3). The difference between the 2B2t-ELISA and HF-ELISA was statistically significant. The most significant cross-reactivity was detected in NCC Peruvian patients for the 2B2t antigen, although two of them also showed a positive immunoblot against HF (data not shown), suggesting that they could actually suffer from CE. Strikingly, no cross-reactivity was noted with the 2B2t-ELISA in 13 NCC Mexican patients (Table 3). The difference in cross-reactivity was statistically significant between the 2B2t-ELISA and each of the other three tests.

**Table 3:** Sensitivity, specificity and cross-reactivity of four different diagnostic tools.

	HF-ELISA	B2t-ELISA	2B2t-ELISA	IHA
**CE Confirmed cases, n = 186 (Sensitivity)**	155 (83.3%*)	147 (79.0%*)	163 (87.6%)	65 (34.9%*)
**Healthy donors, n = 110 (Specificity)**	5 (95.4%*)	1 (99.1%)	1 (99.1%)	0 (100%)
**Cross Reactions (n = 174)**				
AE (n = 57)	57 (100%)	7 (12.3%)	10 (17.5%)	24 (42.1%)
NCC Mexico (n = 13)	7 (53.8%)	1 (7.7%)	0 (0%)	0 (0%)
NCC Peru (n = 57)	22 (38.6%)	14 (24.6%)	21 (36,8%)	1 (1.7%)
Taeniasis Peru (n = 11)	4 (36.4%)	1 (9.1%)	2 (18.2%)	0 (0%)
Hepatitis (n = 36)	0 (0%)	1 (2.8%)	1 (2.8%)	1 (2.8%)
Overall cross-reactions (n = 174)	90 (51.7%*)	24 (13.8%*)	34 (19.5%)	26 (14.9%*)

The diagnostic performance of the tools B2t-, 2B2t- and hydatid fluid (HF)-ELISA, and a commercial indirect haemagglutination test (IHA), were compared using a panel of sera from cystic echinoccocosis (CE) patients, healthy donors and patients with other diseases (see below). The results are shown as the number of positive results against the different tools and the corresponding sensitivity, specificity and cross-reactivity for each group. Under “Cross Reactions”: AE: alveolar echinococcosis; NCC MEX: neurocysticercosis Mexico; NCC PERU: neurocysticercosis Peru. n: number of samples.

*: indicates significant differences between the HF-ELISA, B2t-ELISA or IHA tests and the 2B2t-ELISA.

Several variables that could potentially affect the sensitivity of the tests with the different in-house antigens and the commercial IHA kit were analysed. These variables included the number of cysts (one or more), localization (liver or lung), WHO ultrasound classification (liver cysts were grouped into two subgroups, i.e., CE1, and CE2 to CE4, to have a representative number of cases in each subgroup), CE complications (ruptured cyst, in 51 patients, or infected cyst, in 47 patients), CE antecedents and date of serum collection *vs*. date of treatment onset (of the 57 patients whose serum was collected after treatment onset, 55 were surgically treated and two were only treated with albendazole). First, a chi squared test was used to calculate the sensitivity of the tests for each variable, without considering the potential influence of the others. The results from this analysis are shown in Table 4. The sensitivity of the test with the HF antigen was shown to be affected by a higher number of variables as compared to the recombinant antigens, including the number of cysts and their localisation and classification, CE antecedents, complications and the collection of serum before or after treatment. Similarly, the results obtained with the IHA kit were affected by all variables, except for CE antecedents. The sensitivity of the test with the B2t antigen was affected by the number of cysts and the date of serum collection. Indeed, the screening of patients with a single cyst was more frequently negative than that of patients with more multiple cysts, and serum collection after treatment was associated with an increased probability of presenting a positive result by B2t-ELISA. The performance of the 2B2t recombinant protein was only affected by one variable (the number of cysts), and showed a lower number of positive results in patients with a single cyst than in those with multiple cysts (*P*<0.05). The time of serum collection, i.e., before or after surgical treatment, which affected the performance of the test using the B2t antigen, did not affect the performance of the test using the 2B2t antigen. However, the influence of other treatments should be further investigated. A higher number of positive patients was consistently detected with the 2B2t than with the HF or B2t antigens in all groups.

**Table 4:** Influence of clinical variables on the diagnostic sensitivity of four different serodiagnostic tools (chi^2^).

		HF-ELISA		B2t-ELISA		2B2t-ELISA		IHA	
		Positive	*P*	Positive	*P*	Positive	*P*	Positive	*P*
**NUMBER OF CYSTS**	**Single (n = 108)**	85 (78.7%)	**0.046**	78 (72.2%)	**0.007**	90 (83.3%)	**0.036**	26 (24.1%)	**0.000**
	**Multiple (n = 78)**	70 (89.7%)		69 (88.5%)		73 (93.6%)		39 (50.0%)	
**CYST LOCALISATION**	**Liver (n = 65)**	48 (73.8%)	0.060	49 (75.4%)	0.716	57 (87.7%)	0.385	11 (16.9%)	**0.002**
	**Lung (n = 86)**	74 (86.0%)		67 (77.9%)		71 (82.5%)		35 (40.7%)	
**WHO CLASSIFICATION** *	**CE1 (n = 44)**	29 (65.9%)	**0.012**	31 (70.4%)	0.078	39 (88.6%)	1.000	6 (13.6%)	**0.032**
	**CE2 to CE4 (n = 32)**	29 (90.6%)		28 (87.5%)		29 (90.6%)		11 (34.4%)	
**COMPLICATIONS**	**No (n = 87)**	65 (74.7%)	**0.003**	65 (74.7%)	0.186	74 (85.0%)	0.330	18 (20.7%)	**0.000**
	**Yes (n = 98)**	89 (90.8%)		81 (82.6%)		88 (89.8%)		46 (46.9%)	
**FIRST CE**	**Yes (n = 20)**	20 (100%)	**0.028**	17 (85.0%)	0.771	19 (95.0%)	0.476	10 (50.0%)	**0.135**
	**No (n = 166)**	135 (81.3%)		130 (78.3%)		144 (86.7%)		55 (33.1%)	
**SERUM COLLECTION**	**Before tr. (n = 129)**	100 (77.5%)	**0.001**	96 (74.4%)	**0.020**	111 (86.0%)	0.322	35 (27.1%)	**0.001**
	**After tr. (n = 57)**	55 (96.5%)		51 (89.5%)		52 (91.2%)		30 (52.6%)	

The diagnostic sensitivity of hydatid fluid (HF)-, B2t- and 2B2t-ELISA, and a commercial IHA kit were compared regarding different clinical variables in a panel of cystic echinococcosis patients. Differences are considered significant if *P*≤0.050 (in bold). The results are shown as the number of positive samples and corresponding sensitivity (%) against the different antigens.

*: refers to liver cysts only. tr: treatment.

The influence of complications was also assessed by separating the complicated group into two subgroups, i.e., those with ruptured cysts or infected cysts. Among the 98 patients with complications, 51 (52%) had ruptured cysts and 47 (48%) had infected cysts. The difference in the number of positive results between the two subgroups was not statistically significant for any of the antigens tested (data not shown).

The results of the bivariate analysis, which was used to compare the results obtained with each test and to assess the influence of different clinical variables are shown in Table 5. The IHA test was the one affected by the highest number of variables. Indeed, the presence of more than one cyst, of complications, of cysts in the lungs, and serum collection after treatment were significantly associated with a higher probability of positive result with the IHA test. This higher probability was also observed by multivariate analysis (*P*<0.05; Table 5). On the other hand, CE2 to CE4 cysts, CE complications and serum collection after treatment were significantly associated with a higher probability of positive result with the HF-ELISA. These associations were maintained in the multivariate analysis (*P*<0.05; Table 5). Regarding the recombinant antigens, a higher probability of positive result was noted in the presence of multiple cysts (for B2t and 2B2t) and serum collection after treatment (for B2t; Table 5). However, only the presence of multiple cysts remained statistically significant in the multivariate analysis for B2t (*P*<0.05), but not serum collection after treatment (*P* = 0.053). In addition, the presence of multiple cysts was not associated with a higher probability of positive result with 2B2t (*P* = 0.064).

**Table 5:** Influence of defined clinical variables on the results of four different diagnostic tools (logistic regression).

		HF-ELISA		B2t-ELISA		2B2t-ELISA		IHA	
		B-OR (IC)	M-OR (IC)	B-OR (IC)	M-OR (IC)	B-OR (IC)	M-OR (IC)	B-OR (IC)	M-OR (IC)
**NUMBER OF CYSTS**	**Single**	1	--	1	1	1	1	1	1
	**Multiple**	2.4 (1.0–5.6)	--	**2.9 (1.3–6.6)**	**2.7 (1.2–6.2)**	**2.92 (1.03–8.24)**	2.7 (0.9–7.7)	**3.1 (1.7–5.9)**	**3.0 (1.5–5.9)**
**CYST LOCALISATION**	**Liver**	1	--	1	--	1	--	1	1
	**Lung**	2.2 (1.0–5.0)	--	1.1 (0.5–2.5)	--	0.66 (0.26–1.70)	--	**3.4 (1.5–7.3)**	**2.7 (1.1–6.8)**
WHO CLASSIFICATION*	**CE1**	1	1	1	--	1	--	1	--
	**CE2 to CE4**	**4.0 (1.0–15.5)**	**6.6 (1.1–38.5)**	2.2 (0.6–8.0)	--	1.05 (0.23–4.83)	--	2.2 (0.6–8.2)	--
**COMPLICATIONS**	**No**	1	1	1	--	1	--	1	1
	**Yes**	**3.3 (1.4–7.7)**	**2.4 (1.0–5.8)**	1.6 (0.8–3.3)	--	1.55 (0.64–3.73)	--	**3.4 (1.8–6.5)**	**2.9 (1.5–5.9)**
**FIRST CE**	**Yes**	1	--	1	--	1	--	1	--
	**No**	3.7 (0.0)	--	1.6 (0.4–5.6)	--	2.90 (0.37–22.78)	--	2.0 (0.8–5.1)	--
**SERUM COLLECTION**	**Before tr.**	1	1	1	1	1	--	1	1
	**After tr.**	**8.0 (1.8–34.7)**	**6.6 (1.5–29.1)**	**2.9 (1.1–7.4)**	2.6 (1.0–6.7)	1.69 (0.59–4.79)	--	**3.0 (1.6–5.7)**	**2.6 (1.3–5.1)**

The association and influence of the clinical variables enumerated in the first column on the sensitivity of the hydatid fluid (HF)-, B2t- and 2B2t-ELISAs and of a commercial IHA kit was statistically assessed. B-OR: odds ratio in the bivariate, and M-OR in the multivariant regression analysis. IC: 95% confidence interval. Statistically significant differences (*P*≤0.050) are shown in the table (in bold). tr.: treatment.

## Discussion

Our findings show that the new 2B2t-ELISA performs better than that of the HF- and B2t-ELISA, and also than that of a commercial haemagglutination test. As both the in-house HF-ELISA and the IHA commercial kit are frequently used for the serodiagnosis of CE patients in clinical settings, the 2B2t-ELISA could represent a good alternative as a routine test for CE serology.

The previously described B2t recombinant antigen [10] was used as the base for constructing the three tandem repeat antigens constituted by two, four and eight subunits. This approach was chosen based on the results of other studies showing an increase in antigenicity when two or more subunits of a specific antigen were cloned together and used for the diagnosis of specific diseases (e.g., leishmaniasis and trypanosomiasis) [11], [13]. This increase in reactivity correlated with the increase in number of repeats in the recombinant proteins studied, and that was also shown here for the 2B2t antigen compared with the B2t antigen. In the present study, surprisingly, the 4B2t antigen was revealed to be less reactive than the recombinant 2B2t antigen, although the latter contained a lower number of B2t subunits than the 4B2t antigen. This could be due to a conformation of the 4B2t protein unfavourable for antibody binding, compared to that of the 2B2t antigen. Indeed, the three-dimensional structure could have resulted in the loss of some conformational epitopes needed for antibody binding.

The higher number of antigenic units also resulted in a lower specificity for the 4B2t-ELISA, which presented a high number of false-positive results in healthy donors than the B2t-ELISA, while the 2B2t-ELISA showed the same specificity than the B2t-ELISA. This loss of specificity associated with the increase in the number of antigenic repeat units has been shown before for other recombinant antigens (e.g., [13]). The cross-reactivity of the 2B2t antigen increased more markedly, as compared to that of the B2t antigen when using serum from Peruvian patients with NCC. The western blot against 8, 16 and 21 kDa HF antigens was only positive in one of the cross-reactive Peruvian patients, and no correlation was found between a higher number of positive bands in the NCC-specific western blot and 2B2t cross-reactivity. Nevertheless, the absence of cross-reactivity in NCC patients from Mexico seemed to indicate the potential presence of true anti-*E. granulosus* antibodies in the NCC Peruvian samples. This could be explained by higher human prevalence of CE (5.5–9.1% in endemic villages) [14], than in Mexico. In fact, autochthonous Mexican CE cases are regarded as rare [15]. Because the serum donors were recruited in an area with a low endemicity for CE (Salamanca, Spain), the potential cross-reactivity detected in Peruvian patients should be further investigated with a panel of donors from the same area as the NCC patients.

The commercial IHA kit proved to be highly specific, but a high cross-reactivity was detected with the serum from AE patients. The cross-reactivity of antigens usually used for the serodiagnosis of CE with alveolar echinococcosis (AE) is a serious drawback for the specific diagnosis of CE in areas where both parasites co-exist [8], [9]. In this regard, the ability of the new 2B2t antigen to discriminate between patients with CE and AE is very interesting.

In spite of the higher sensitivity of the 2B2t-ELISA, which was the best of the four tests assessed here, a percentage of CE confirmed that patients from our cohort still showed negative results with the 2B2t-ELISA. Frequently, studies intending to validate serodiagnostic tools for CE fail to consider several clinical variables that could affect the diagnostic performance of these tools. In addition, serum selection is sometimes based on previous serologic information. As a consequence, divergent results are reported for the same test, e.g., ranging from 45 to 93% with the same recombinant antigen B2 in IgG-ELISA [9]. False-negative results could then be due to clinical features influencing the level of specific circulating antibodies against defined antigens [4]. This has been shown for several variables; among them, the date of serum collection (relative to treatment initiation), as both pharmaceutical and surgical treatments can lead to a rise in the level of specific antibodies (e.g., [16]), the cyst stage (e.g., [17]), cyst localization (e.g., [18]) and the number of cysts (e.g., [19], [20]).

Here, we examined the influence of six different clinical variables on the performance of the tests: the number of cysts, cyst localization, cyst stage, the presence of complications, CE antecedents, and date of serum collection (compared to that of treatment onset). Regarding cyst classification, because the number of samples in our study for some types of cysts was too low for statistical analysis (44 patients with CE1, 22 patients with CE2, eight patients with CE3 and two patients with CE4), especially those belonging to the inactive (CE4-CE5) group, we could not group our patients into active, transitional and inactive groups in the attempt to relate cyst activity with the corresponding serological result. In this regard, the shift from active to inactive cysts has been shown to result in a decline of specific antibodies against the recombinant antigen B (e.g., [21]). Also related to this, the expression of the antigen B2 subunit has been shown to reduce with the senescence of cysts [22]. Therefore, we are aware that the performance of our antigens should be further investigated in a larger group including a higher number of patients with CE3, CE4 and CE5 cysts, preferably in a prospective follow-up trial. Nevertheless, CE1 cysts have also been shown to be more frequently associated with a seronegative response against parasite antigens, including antigen B, in contrast with the CE2 and CE3 types of cysts that are usually associated with a detectable and specific antibody response (e.g., [17], [21]). Thus, we decided to group our patients into two groups: CE1 and CE2 to CE4, and determine if the results of the tests depended on this variable.

All variables studied here (except one) influenced the sensitivity of the HF-ELISA, as observed by chi squared analysis. A very similar result was noted with the IHA commercial test. The B2t-ELISA was influenced by the number of cysts and date of serum collection (compared to that of treatment onset), while the 2B2t-ELISA was only influenced by the number of cysts.

Strikingly, patients with cysts in the lungs were more frequently positive with the IHA kit and HF-ELISA than patients with cysts in the liver. This contradicts the reports of other authors, who showed that cysts in the lungs usually result in a lower antigenic stimulation than cysts in the liver (e.g., [18]). Nevertheless, these differences could be attributed to the influence of other clinical variables. In our group of patients, 66% of cysts in the lungs led to complications, compared to 27% for cysts in the liver, in both localizations at 1∶1 proportion regarding ruptured and infected cysts. Then, the presence of complicated cysts could specifically account for a higher seropositivity influencing the test results, as noted with the IHA kit and HF-ELISA. These observations prompted us to perform a multivariate statistical analysis to assess the influence of all variables together.

The results of the logistic regression indicated that four and three of six variables affected the outcome of the IHA kit and HF-ELISA, respectively, in association or not with the other variables. The results of the B2t-ELISA were influenced by the number of cysts. Thus, a patient with a single cyst is more likely to test negative at B2t than a patient with multiple cysts. On the other hand, the performance of the 2B2t-ELISA was statistically independent from all the clinical variables evaluated. Thus, the percentage of negative patients with a confirmed CE could be attributed to the absence of specific antibodies associated with variables not evaluated in this study, either clinical or other, e.g., the formation of immune complexes.

Nevertheless, although the better sensitivity of the 2B2t antigen over that of B2t resulted in a loss of statistically significant difference for some clinical variables, we are aware that the inclusion of a higher number of patients could result in a statistically significant association for some of the variables tested, especially for those showing a broad 95% confidence interval. Thus, our results suggest that multiple cysts, localisation in the liver *vs*. the lung, CE2 and CE3 cysts or complications, and serum collection after treatment onset are variables that increase the probability of a positive result with the 2B2t-ELISA.

Considering the patients who tested negative with the 2B2t-ELISA (n = 23), 78% had a single cyst, 65% had cysts in the lungs, 62.5% had CE1 types of cysts, 56% suffered from complications, and 78% provided a sample before initiation of the treatment. Unfortunately, the number of patients with negative serology was too low to draw any significant conclusion. Nevertheless, we could assume that a patient with an ultrasound image suggestive of CE, but presenting a single CE1 uncomplicated cyst located in the lungs, and whose was taken prior to the administration of any treatment, would probably yield a false-negative result with the 2B2t-ELISA.

A striking example of how the results of a given test can change depending on the characteristics of a patient, was shown in the high difference in specificity observed for the B2t-ELISA between the present study and the assessment performed previously [10]. In this previous study, the serum of 102 Spanish CE patients was analyzed, and a sensitivity of 91.2% was obtained with the B2t-ELISA. When the new 2B2t antigen was tested using the serum of these 102 patients, a sensitivity of 94.1% was obtained (data not shown). Unfortunately, the clinical data available from these patients was scarce. Thus, this higher sensitivity cannot be attributed to specific variables. Similarly, the results obtained by other authors using the recombinant antigen B2 for the detection of specific IgG in ELISA show highly variable sensitivities, ranging from 45% to 93% [9]. From these studies, the sensitivities reported by Rott et al. [23] and Virginio et al. [24] are the most similar to the sensitivity found here for the B2t and 2B2t recombinant antigens. Unfortunately, further comparison between our results and those obtained in these studies is difficult, as previously discussed [10], for a number of reasons, among which missing clinical information on the patients used by these authors. Nevertheless, these differences indicate the need to reach a consensus to determine the variables to be checked when using a serodiagnostic tool for CE. In addition, it is best to use the same samples to compare different serological tools [25]. Short of this, there are few chances that a better test to detect specific antibodies in CE patients, which could replace the very variable and clearly improvable tests based on the use of native antigens, can be developed.

In spite of its limititations, to our knowledge this is the first attempt to take into account some of the variables of the extremely complex clinical presentation of human CE [26] in the evaluation of several serological tests. T We also show that the performance of a serological test with a defined and specific recombinant antigen can be improved by using two units of that antigen in a tandem construct.
